# Effective training procedure for a simultaneous bimanual movement task in head-fixed mice

**DOI:** 10.3389/fncir.2025.1633843

**Published:** 2025-08-08

**Authors:** Kotaro Tezuka, Hironobu Osaki, Kaneyasu Nishimura, Shin-Ichiro Terada, Masanori Matsuzaki, Yoshito Masamizu

**Affiliations:** ^1^Laboratory of Functional Brain Circuit Construction, Graduate School of Brain Science, Doshisha University, Kyoto, Japan; ^2^Department of Physiology, Graduate School of Medicine, The University of Tokyo, Tokyo, Japan; ^3^Brain Functional Dynamics Collaboration Laboratory, RIKEN Center for Brain Science, Saitama, Japan; ^4^International Research Center for Neurointelligence (WPI-IRCN), The University of Tokyo Institutes for Advanced Study, Tokyo, Japan; ^5^Department of Biological Sciences, Graduate School of Science, The University of Tokyo, Tokyo, Japan

**Keywords:** simultaneous bimanual movements, forelimb movements, motor task, motor control, lever-pull, head-fixed

## Abstract

Bimanual movements consist of simultaneous and nonsimultaneous movements. The neural mechanisms of unimanual and nonsimultaneous bimanual movements have been explored in rodent studies through electrophysiological recordings and calcium imaging techniques. However, the neural bases of simultaneous bimanual movements remain poorly understood because of a lack of effective training procedures for such movements in head-fixed rodents. To address this issue, we developed a task in which mice simultaneously pull right and left levers with their forelimbs in a head-fixed condition. Here, we conducted sessions with the link plate in which both levers were mechanically linked to help mice learn the importance of simultaneous bimanual movements. These sessions with the link plate enabled the mice to maintain high success rates even during independent sessions, where the right and left levers could move independently. In these independent sessions, mice were not required to pull both levers at the same time, but rather simply to hold levers simultaneously for a specific period. The mice that experienced sessions with the link plate showed a significantly higher ratio of simultaneous (i.e., lag < 20 ms) than nonsimultaneous lever pulls. In contrast, mice without experience in sessions with the link plate showed no significant increase in simultaneous over nonsimultaneous pulls. This study demonstrates the efficacy of our new task in facilitating repetitive simultaneous forelimb movements in rodents and provides a basis for understanding the neural mechanisms underlying bimanual movements.

## 1 Introduction

Bimanual movement is the ability to coordinate both hands and is essential for performing numerous tasks in our daily lives. From simple activities, like eating and dressing, to increasingly more complex actions, the coordination of both hands is essential for movement efficiency and accuracy. Various activities require different motor elements, such as unimanual and bimanual, as well as simultaneous and nonsimultaneous movements.

Research on hand movement in primates has shown that the supplementary motor area (Tanji et al., 1987, 1988; Sadato et al., 1997; Kazennikov et al., 1999; Aramaki et al., 2010), dorsal premotor area (Sadato et al., 1997; Kermadi et al., 2000; Aramaki et al., 2010), and primary motor cortex (M1) (Donchin et al., 1998; Kazennikov et al., 1999; Cross et al., 2020) are engaged during bimanual movement tasks. The interaction between these areas in both cortical hemispheres via the corpus callosum is also crucial for bimanual movement, as demonstrated in studies on primates (Brinkman, 1984; Cardoso de Oliveira et al., 2001) and humans (Meyer et al., 1995; Meyer et al., 1998; Eliassen et al., 1999; Franz et al., 2000; Kennerley et al., 2002; Diedrichsen et al., 2003; Sternad et al., 2007; Bonzano et al., 2008; Gooijers and Swinnen, 2014). Thus, understanding the neural mechanisms underlying bimanual movement requires the examination of multiple areas across both hemispheres.

Rodents are ideal models for exploring the various brain regions involved in bimanual movement using techniques such as two-photon calcium imaging and electrophysiological methods (Jordan et al., 2024). These approaches enable the simultaneous examination of multiple areas in both hemispheres. Furthermore, the use of head-fixed conditions helps suppress motion artifacts and facilitates stable behavioral monitoring and neural recording (Guo et al., 2014). Several tasks have been developed to take advantage of these benefits under head-fixed conditions. For example, under head-fixed conditions, unimanual movements have been investigated using lever-pulling tasks (Isomura et al., 2009; Hira et al., 2013; Masamizu et al., 2014; Terada et al., 2022), while nonsimultaneous bimanual movements have been studied using pedal-pushing tasks (Soma et al., 2017; Jeong et al., 2021). Therefore, revealing and comparing the neural activities of each movement element would facilitate understanding of the information-processing mechanisms of complex movement.

Despite these advancements, the neural mechanisms underlying simultaneous bimanual movement remain largely unknown. One of the primary challenges is the lack of an effective training procedure for simultaneous bimanual movement tasks in head-fixed rodents. In this study, we developed a new experimental system in which mice pull the right and left levers simultaneously with their bilateral forelimbs in a head-fixed condition, improving on a previously developed unimanual lever-pull task (Hira et al., 2013). The proposed system represents a first step toward obtaining a more rigorous understanding of the neural mechanisms of simultaneous bimanual movement.

## 2 Materials and methods

### 2.1 Animals and surgery

All animal experiments were approved by the Institutional Animal Care and Use Committee of Doshisha University. Eleven male C57BL/6 mice were used for the experiments. The mice were all raised in home cages under a 12-h light/dark cycle, and all experiments were conducted during the light phase.

For the surgical procedure, each animal was anesthetized by intraperitoneal injection of an anesthetic mixture of medetomidine (0.3 mg/kg, Domitor, Nippon Zenyaku Kogyo, Fukushima, Japan), midazolam (4.0 mg/kg, Dormicum, Maruishi Pharmaceutical, Osaka, Japan), and butorphanol (5.0 mg/kg, Vetorphale, Meiji Animal Health, Kumamoto, Japan). Ampicillin (80 mg/kg, Ampicillin Sodium NZ, Nippon Zenyaku Kogyo, Fukushima, Japan) and carprofen (6.0 mg/kg, Rimadyl, Zoetis Japan, Tokyo, Japan) were subsequently administered intraperitoneally. Eye ointment (Tarivid, Santen Pharmaceutical, Osaka, Japan) was applied to prevent eye dryness. The skin of each mouse head was disinfected with chlorhexidine gluconate solution (Hibitane Solution, Sumitomo Pharma, Osaka, Japan), and the fur was shaved with a razor blade (FA-10, Feather Safety Razor, Osaka, Japan). Transdermal lidocaine (Xylocaine Jelly, Sandoz Pharma, Tokyo, Japan) was then applied to the skin, and an incision was made. After removing the periosteum, the neck muscle was cut approximately 2 mm rostrally along the midline to provide space for the head plate. The head plate (Supplementary CADdata; Tsukasa Giken, Shizuoka, Japan) was firmly attached to the skull using dental cement (Estecem II, Tokuyama Dental, Tokyo, Japan), as described previously (Hira et al., 2013). The surface of the intact skull was coated with dental resin cement (Super Bond, Sun Medical, Shiga, Japan). After surgery, the medetomidine antagonist atipamezole (0.3 mg/kg, Antisedan, Nippon Zenyaku Kogyo, Fukushima, Japan) was administered intraperitoneally to revive the mice from the anesthetic state. After head plate attachment, the mice were allowed to recover for at least 3 days before any further procedures.

### 2.2 Water restriction

Water was restricted to motivate the mice to pull the lever. During the rest period, the mice were allowed to drink water freely from bottles installed in the home cage. Two days prior to training, the bottles were removed, and water restrictions were initiated. The mice were maintained at 80–85% of their pretraining body weight. If the amount of water given during each session did not reach 1 ml, the remaining volume was added after the session to maintain body weight above 80%. The remaining volume of water was provided via a 100 ml conical tube (2355–100, IWAKI, Tokyo, Japan) placed in the home cage with its opening facing upward and tilted at an angle of approximately 10 degrees from horizontal. On days when training was not conducted, an agar block containing water was placed in a 100 ml conical tube in the home cage to maintain body weight above 80% of its pretraining value.

### 2.3 Sound cue-triggered simultaneous bimanual lever-pull task

**Figure 1:**
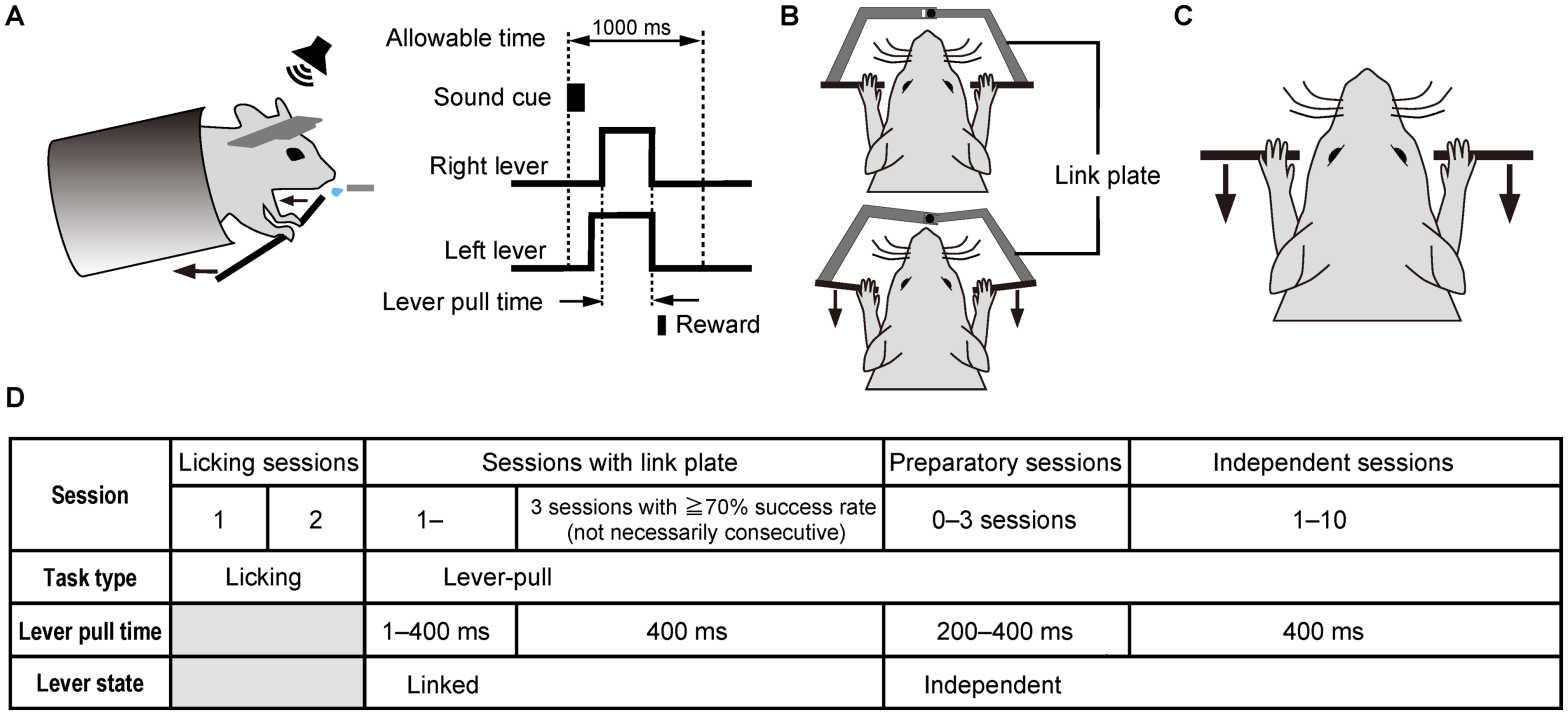
The simultaneous bimanual lever-pull task performed after a sound cue using both forelimbs. **(A)** Schematic diagram of the sound cue-triggered simultaneous bimanual lever-pull task. Head-fixed mice are trained to pull the right and left levers within 1 s (allowable time) after the onset of the 100-ms sound cue. The lever-pull time is defined as the interval during in which both the right and left levers are both pulled to obtain the reward. **(B)** Linked state of the right and left levers, in which they are linked by a link plate, causing them to be moved simultaneously. Top, the state in which the right and left levers are not pulled; bottom, the state in which the right and left levers are pulled. **(C)** Independent lever state of the right and left levers, in which each lever can be moved independently. **(D)** Training schedule of the sound cue-triggered simultaneous bimanual lever-pull task.

The task apparatus consisted of lever units and a link plate (OPR-LU-MJ, O’Hara & Co., Tokyo, Japan), a head plate holder (OPR-3702MA, O’Hara & Co., Tokyo, Japan), a body chamber (OPR-MAΦ23, O’Hara & Co., Tokyo, Japan), a variable angle unit for the body chamber (OPR-BFAM, O’Hara & Co., Tokyo, Japan), a licking sensor (OPR-LKMA, O’Hara & Co., Tokyo, Japan), a sound stimulation unit (OPR-SSSMR, O’Hara & Co., Tokyo, Japan), and a water supply unit (OPR-7300, O’Hara & Co., Tokyo, Japan). We developed a sound cue-triggered simultaneous bimanual lever-pull task that was a modification of a previously described sound cue-triggered unimanual lever-pull task (Terada et al., 2018). In this task, head-fixed mice were trained to respond to a 100-ms (11 kHz sinusoidal tone, 85–90 dB) sound cue within a 1-s time window by simultaneously pulling the right and left levers, which were placed 5 mm apart, simultaneously using their forelimbs. The maximum displacement of the levers was 4 mm. The mice had to hold both levers simultaneously at over 40% of the maximum displacement for a duration of 400 ms, to obtain 4 μl of water as a reward (Figure 1A). The valve-open duration of the water supply unit is below 200 ms. The sound cue was presented through a speaker (FT28D, Fosterx, Tokyo, Japan) on the right side, which was placed 15 cm from the animal. The sound cue was repeated at 2.5- to 3.5-s intervals after the levers were returned to their starting positions. When the mouse pulled the lever outside of the 1-s time window, an additional delay of 2.5–3.5 s intervals was imposed after the levers regained to their starting positions.

In the current task, the body chamber, which was positioned horizontally in the unimanual lever-pull task (Hira et al., 2013), was tilted 20 degrees, elevating the rostral side, to prevent the mice from pushing the levers (Figure 1A). In initial lever-pulling training sessions (sessions with the link plate), the right and left levers were mechanically linked using a link plate to ensure that the mice learned to pull them simultaneously (Figure 1B), mitigating the likelihood of asynchronous pulling after transitioning to an independent lever state (Figure 1C). The link plate consists of two separate plates, each of which can be attached to the right and left levers. These plates were designed to be connected at their ends via a ball bearing, allowing the right and left levers to move synchronously when attached to the lever shaft (Supplementary Figure 1).

To train the mice to perform the sound cue-triggered bimanual lever-pull task, the following procedure was implemented (Figure 1D): In the first and second sessions (licking sessions), the mice were given 4 μl of water as a reward when they licked the spout within the 1-s time window after the onset of the 100-ms sound cue, thereby associating the sound cue with the reward. The reward timing was controlled by the licking sensor. Each mouse was trained to perform the licking task for 30 min per day for two consecutive days. In the first and second licking sessions, the mean success rates were 78% ± 9.7% and 86% ± 15.0% (mean ± SD, *n* = 7), respectively, with 124 ± 35.3 and 129 ± 64.0 (mean ± SD) successful attempts, respectively, and with 159 ± 40.2 and 166 ± 106.5 (mean ± SD) trial number, respectively. From the next session, bimanual lever-pull training was initiated. Initially, both levers moved together (linked lever state), and the lever pull time required to obtain the reward was gradually extended from 1 ms to 400 ms by the fifth session with the link plate. Here, the lever pull time refers to the interval during which the mouse was required to continuously pull the lever to receive the reward. In this setting, the mouse obtained the reward when the pull duration reached a preset threshold. The gradual extension of the lever pull time was achieved through an automated control system: the required pull time increased by 50 ms after each successful trial, while it was simultaneously reduced by 1 ms every 400 ms, within the range between the minimum and maximum lever pull time defined by the experimenter. The automated control system continued to be used until the lever pull time reached 400 ms. After this training, the lever pull time was fixed at 400 ms. Once the success rate exceeded 70% for three sessions in the linked state (not necessarily consecutive), the link plate was removed to allow independent movement of each lever (independent lever state). The mean number of sessions with the link plate was 7.7 ± 2.2 sessions (mean ± SD, *n* = 7). For up to three sessions after the ones with the link plate, the pull time duration was temporarily shortened so that the mice could adapt more easily to the independent lever state (preparatory sessions). At this session, the automated control system for extending pull time extension was also used until it reached 400 ms. After these preparatory sessions, independent sessions were initiated, with the lever pull time fixed at 400 ms. The mice needed to pull the right and left levers separately within the 1-s time window to obtain the reward. Each daily session was at least 30 min long. After 30 min, the session was terminated when the number of successful attempts reached 250, the mouse stopped pulling the levers, or the session’s duration reached 40 min.

For mice that did not experience the sessions with the link plate, all training procedures were the same as for mice that experienced the session with the link plate, except the levers were not linked from the initial lever-pull task (Supplementary Figure 2A).

These processes were controlled by a program written in LabVIEW (National Instruments, TX, USA). The lever position was detected using a rotary encoder (MES-12-2000P, Microtech Laboratory Inc., Kanagawa, Japan) installed at a distance of 8 cm from the lever tip. The pulse output of the rotary encoder was counted with an NI-DAQ (USB-6343, National Instruments, TX, USA), and converted into the arc length, which was recorded with other analog data including lick status from the licking sensor. The lever position was sampled at 1 kHz and subsequently low-pass filtered at 450 Hz for further analysis. To check whether animals grasp both levers during the task, we also monitored the right and left forelimb positions including whole body movement using a CMOS camera (DMK33UP1300, The Imaging Source, Bremen, Germany) controlled by a custom written Matlab program (R2023B, MathWorks, MA, USA).

### 2.4 Analysis

The lever trajectories were recorded as time series data of lever positions. Using these data, the response latency was calculated as the time when both levers were pulled over the displacement threshold after the sound cue. Lag was defined as the time difference between the onset times at which the right and left lever pulls exceeded the displacement threshold.

To compare the lag distribution of mice that experienced the sessions with the link plate to those of mice that did not experience these sessions, we used the data from sessions 13–15. These sessions were counted from the first licking session to determine the session number. As the mice that did not experience the sessions with the link plate were trained according to the schedule shown in Supplementary Figure 2A, we used the data from the sessions in which the lever pull time reached 400 ms (independent sessions) for this comparison. The total trials across sessions were shown in Supplementary Figure 3. One mouse that experienced the sessions with the link plate was excluded from the analysis because it continued the sessions with the link plate at sessions 13–15 counted from the first licking session.

The success rate was calculated by dividing the number of successful attempts by the number of the sound cue number. The success rate, response latency, and number of successful attempts were analyzed using generalized linear mixed models (GLMMs) with a Poisson distribution and log link function, implemented in MATLAB using the fitglme function. The model included Session as the fixed effect and Subject as the random effect. The model structures were as follows:


$$\log({\text{SuccessRate}}_{\mathrm{ij}})=\beta_{0}+\beta_{1}({\text{Session}}_{i})+u_{\mathrm{0j}}+u_{\mathrm{1j}}({\text{Session}}_{i})+\varepsilon_{\mathrm{ij}}$$

$$\log({\text{ResponseLatency}}_{\mathrm{ij}})=\beta_{0}+\beta_{1}({\text{Session}}_{i})+u_{\mathrm{0j}}+u_{\mathrm{1j}}({\text{Session}}_{i})+\varepsilon_{\mathrm{ij}}$$

$$\log({\text{SuccessNumber}}_{\mathrm{ij}})=\beta_{0}+\beta_{1}({\text{Session}}_{i})+u_{\mathrm{0j}}+u_{\mathrm{1j}}({\text{Session}}_{i})+\varepsilon_{\mathrm{ij}}$$

where SuccessRate_ij_, Responselatency_ij_, and SuccessNumber_ij_ represent the expected number of trials, success rate, response latency, and number of successful attempts for subject j on session i, respectively. β_0_ represents the model intercept, β_1_ represents the estimated regression coefficient, u_0j_ represents the random intercept for subject j, u_1j_ represents the random slope for the effect of the session for subject j, and ε_ij_ represents the residual.

Continuous data are presented as mean values with standard deviations (when normally distributed) or median values with interquartile ranges (when non-normally distributed). Categorical data are presented as frequencies with percentages. The mean number of successful attempts per lag interval was analyzed using the one-way analysis of variance, followed by the Tukey–Kramer test to determine significant differences. The success rate was analyzed using Wilcoxon signed-rank test. All data analyses, graphing, and statistical tests were performed using a custom analysis program written in MATLAB.

## 3 Results

### 3.1 Learning of the sound cue-triggered simultaneous bimanual lever-pull task

**Figure 2:**
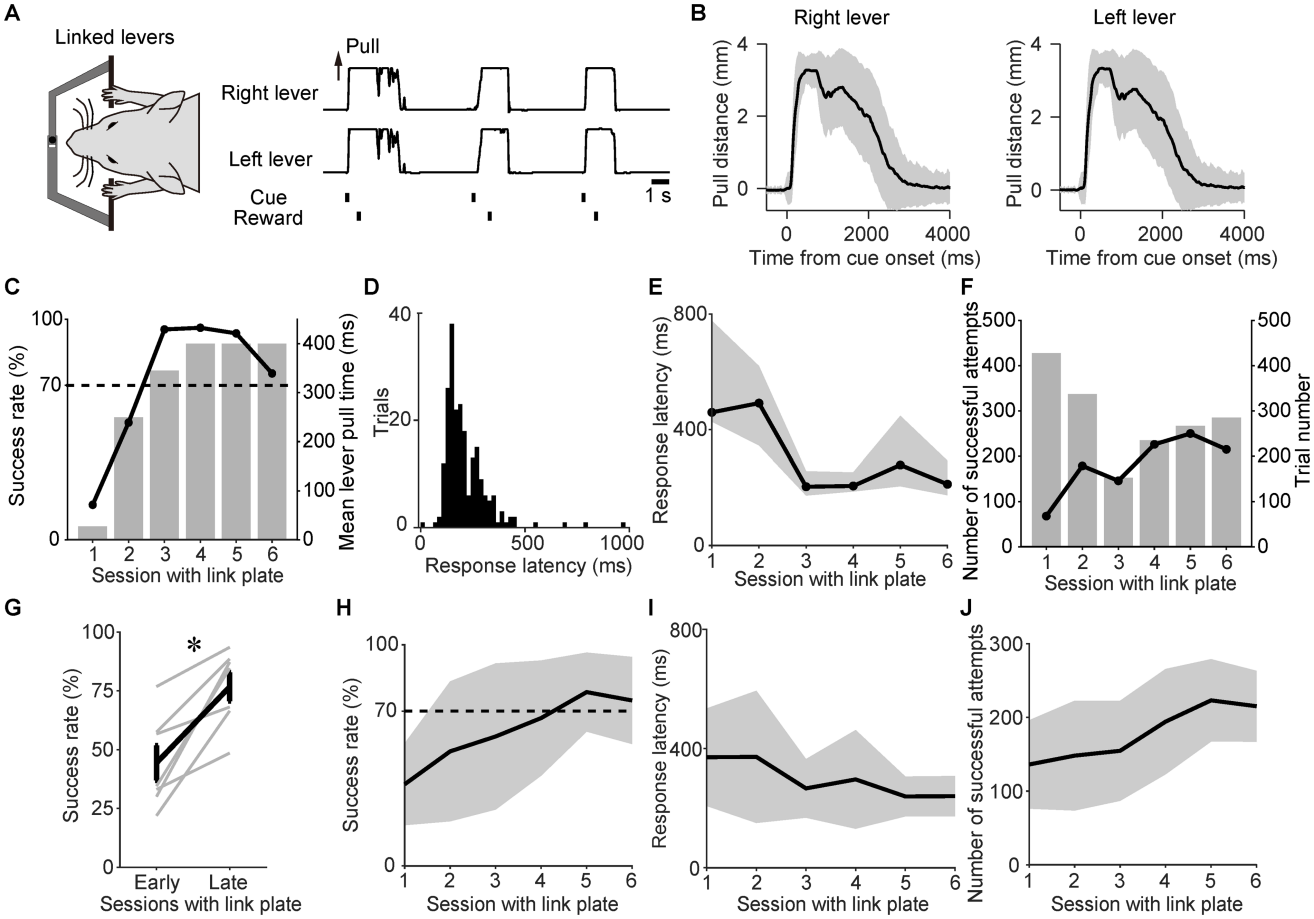
Training mice to perform the sound cue-triggered simultaneous bimanual lever-pull task. **(A)** Example of the right and left lever trajectories from the session with the link plate 6 for a representative mouse. The correlation coefficient between the right and left lever trajectories during the interval from the onset of the sound cue to the reward was 0.99. **(B)** The mean of the right and left lever trajectories during the session with the link plate 6 for the same mouse in **(A)**. The black line represents the mean lever trajectory, while the gray area represents the standard deviation. Lever trajectories were aligned to the onset of the sound cue. **(C)** Changes in success rate for the same mouse in **(A)**. The overlaid bars represent the mean lever-pull time for each session. **(D)** Histogram of response latency for successful trials during the session with the link plate 6 for the same mouse in **(A)**. Each bin width is 20 ms. **(E)** Changes in the response latency for the same mouse in **(A)**. Each value represents the median response latency calculated from histograms, such as **(D)**, while the gray area indicates the interquartile range (25^th^ to 75^th^ percentiles) for each session with the link plate. **(F)** Changes in the number of successful attempts for the same mouse in **(A)**. The overlaid bars represent the trial number for each session. **(G)** Changes in the success rates during sessions with the link plate 1–2 (early) and 5–6 (late). The gray lines indicate individual mice. The bold black line represents the mean success rate among the mice. The success rate differed significantly between the early and late periods (*p* = 0.016, Wilcoxon signed-rank test, *p < 0.05; *n* = 7). **(H)** Changes in the success rate of the population means. The black line and gray area indicate the mean value and standard deviation, respectively. The success rate significantly increased across sessions (GLMM, *p* = $8.5\times 10^{-5}$ ; the estimated regression coefficient (β_1_ ± standard error) was 0.14 ± 0.03; the odds ratio [exp(β_1_)] was 1.15 [95% confidence interval (CI), 1.08–1.23]; the model intercept (β_0_) was 3.54; *n* = 7, see Materials and Methods). **(I)** Changes in the response latency of the population means. The black line and gray area indicate the mean value and standard deviation, respectively. The response latency significantly decreased across sessions [GLMM, *p* = 0.019; β_1_ ± standard error was −0.08 ± 0.03; exp(β_1_) was 0.92 (95% CI, 0.86–0.99); β_0_ was 5.91; *n* = 7]. **(J)** Changes in the number of successful attempts of the population means. The black line and gray area indicate the mean and standard deviation, respectively. The number of successful attempts significantly increased across sessions [GLMM, *p* = $2.3\times 10^{-4}$ ; β_1_ ± standard error was 0.11 ± 0.03; exp(β_1_) was 1.11 (95% CI, 1.06–1.18); β_0_ was 4.73; *n* = 7].

As shown in the task schedule (Figure 1D), the lever-pull task was initiated in the linked state to teach the mice the importance of the simultaneous pulling of both levers in obtaining rewards. After the onset of the sound cue, the mice simultaneously pulled the right and left levers and obtained a reward (Figure 2A). In the linked state, the mean trajectories of each lever for a representative mouse were almost the same (Figure 2B). For this representative mouse, the success rate increased with each successive session, and the high success rates were maintained from the third session with the link plate (Figure 2C). The lever pull time was gradually increased to and, from the fourth session with the link plate, fixed at 400 ms (Figure 2C). In the representative session, the response latency from the onset of the sound cue until the distance between the right and left lever pulls exceeded the displacement threshold was illustrated as a histogram, and the mode was approximately 160 ms (Figure 2D). The response latency decreased with each successive session (Figure 2E), whereas the number of successful attempts increased (Figure 2F). Population data analysis revealed that the success rate increased significantly compared with that in early sessions (Wilcoxon signed-rank test, *n* = 7; Figures 2G,H). The response latency tended to decrease slightly with each session (Figure 2I), whereas the number of successful attempts increased significantly (Figure 2J), confirming acquisition of the task through repeated training. These results indicate that the mice learned the task.

### 3.2 Stability of task performance during the independent lever state

**Figure 3:**
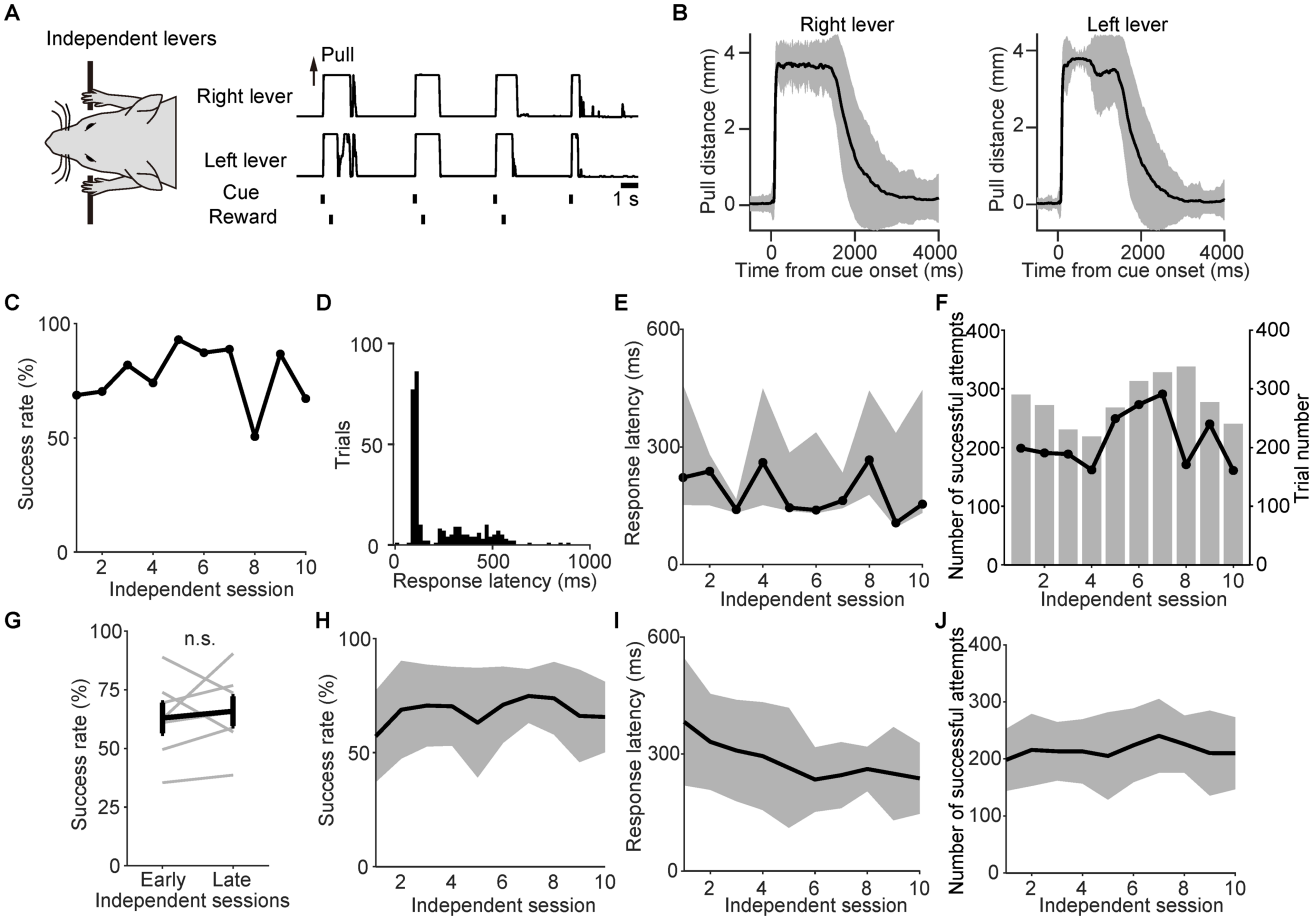
Stable task performance during the independent lever state. **(A)** Example of the right and left lever trajectories from independent session 6 for a representative mouse. The correlation coefficient between the right and left lever trajectories in the interval from the onset of the sound cue to the reward was 0.93. **(B)** The mean value of the right and left lever trajectories from independent session 6 for the same mouse in **(A)**. The black line represents the mean lever trajectory, while the gray area represents the standard deviation. Lever trajectories were aligned to the onset of the sound cue. **(C)** Changes in the success rate for the same mouse in **(A)**. **(D)** Histogram of the response latency in successful trials from independent session 6 for the same mouse in **(A)**. Each bin width is 20 ms. **(E)** Changes in response latency for the same mouse in **(A)**. Each value indicates the median response latency calculated from histograms, such as **(D)**, while the gray area indicates the interquartile range (25^th^ to 75^th^ percentiles) for each independent session. **(F)** Changes in the number of successful attempts for the same mouse in **(A)**. The overlaid bars represent the trial number for each session. **(G)** Changes in the success rate during independent sessions 1–2 (early) and 9–10 (late). Gray lines indicate individual mice. The bold black line indicates the mean success rate among the mice. No significant difference in success rate was observed between the early and late periods (*p* = 0.69, Wilcoxon signed-rank test; *n* = 7). **(H)** Changes in the success rate of the population means. The black line and gray area indicate the mean value and standard deviation, respectively. There was no significant change in success rate across sessions [GLMM, *p* = 0.36; β_1_ ± standard error was 0.01 ± 0.01; exp(β_1_) was 1.01 (95% CI, 0.99–1.03); β_0_ was 4.15; *n* = 7]. **(I)** Changes in the response latency of the population means. The black line and gray area indicate the mean value and standard deviation, respectively. The response latency significantly decreased across sessions [GLMM, *p* = 0.00086; β_1_ ± standard error was −0.05 ± 0.02; exp(β_1_) was 0.95 (95% CI, 0.92–0.99); β_0_ was 5.86; *n* = 7]. **(J)** Changes in the number of successful attempts of the population means. The black line and gray area indicate the mean value and standard deviation, respectively. There was no significant difference in the number of successful attempts [GLMM, *p* = 0.52; β_1_ ± standard error was 0.0051 ± 0.01; exp(β_1_) was 1.0 (95% CI, 0.99–1.02); β_0_ was 5.32; *n* = 7].

In the linked state, the right and left levers were pulled simultaneously because of the link plate, resulting in the passive movement of one lever when the other was pulled. Therefore, we examined whether the mice would still perform the simultaneous bimanual lever-pull movement even after removing the link plate and transitioning to the independent lever state. Thus, we investigated the changes in task performance during the independent lever state. Since the right and left levers could now move independently, some divergence was observed between the individual trajectories of each lever in a representative session, especially after disbursing the reward (Figures 3A,B). However, most of the movements remained similar. Fittingly, the correlation coefficient between the right and left lever trajectories from the onset of the sound cue to the reward was 0.93 (Figures 3A,B). The representative mouse was able to maintain a success rate of approximately 50–93% (Figure 3C). In the representative session, the response latency from the onset of the sound cue until the moment that the distance between the two lever pulls exceeded the displacement threshold was expressed as a histogram, and the mode was approximately 120 ms (Figure 3D). The response latency and number of successful attempts did not increase or decrease with each session (Figures 3E,F). Population data analyses showed no significant difference in success rates between the early and late independent sessions (Wilcoxon signed-rank test, *n* = 7; Figures 3G,H). Although the response latency decreased significantly with each session (Figure 3I), the number of successful attempts did not change significantly over the course of the session (Figure 3J). These results demonstrate that the mice performed the task efficiently and consistently, even after unlinking the right and left levers.

### 3.3 Advantages of experiencing sessions with the link plate

**Figure 4:**
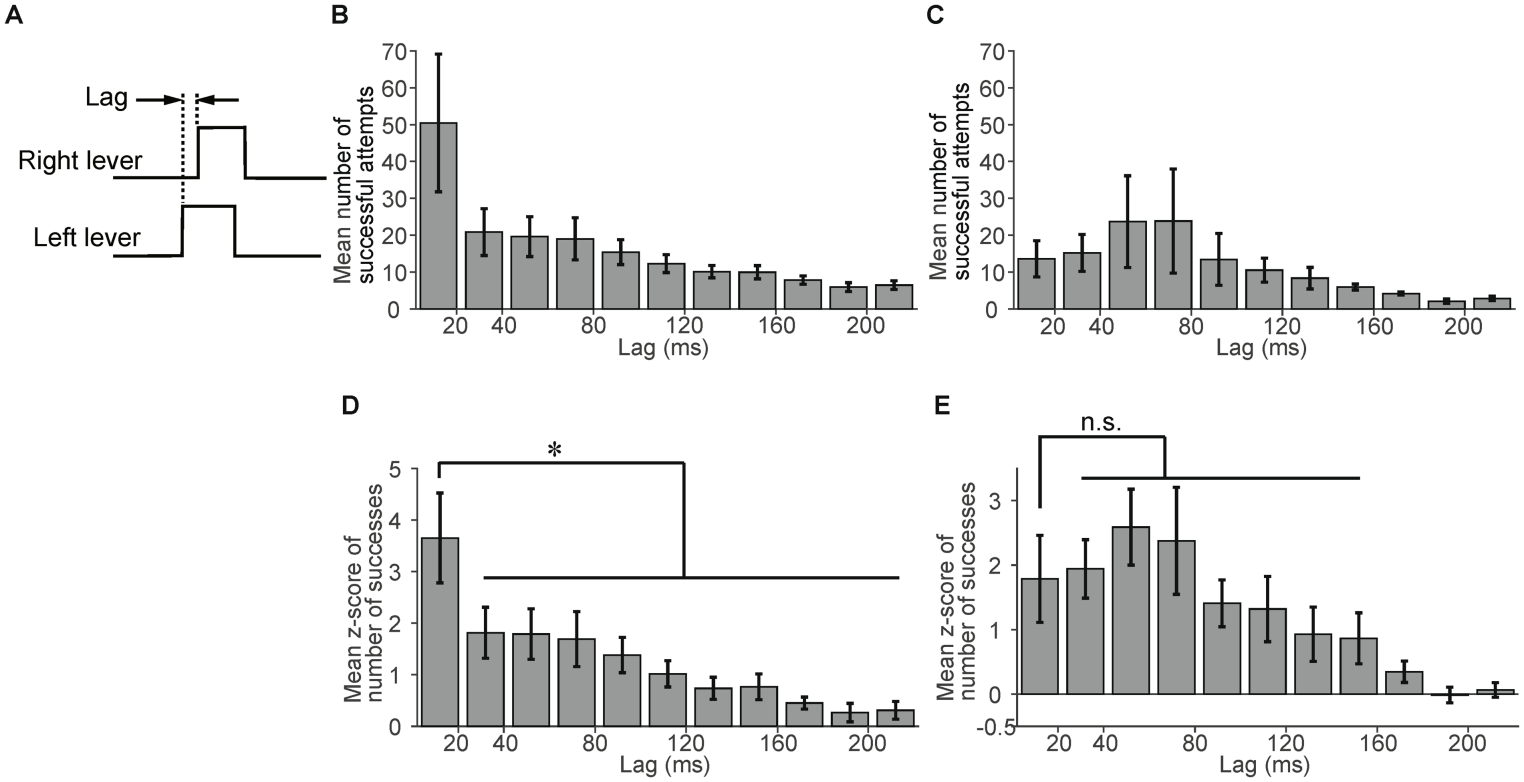
Advantages of experiencing sessions with the link plate. **(A)** Lag was defined as the time difference between the onset of the right and left lever pulls. **(B)** The mean number of successful attempts per lag interval in mice that experienced sessions with the link plate (*n* = 6). Each bin width is 20 ms. The error bars indicate the standard error of the mean. **(C)** The mean number of successful attempts per lag interval in mice that did not experience sessions with the link plate (*n* = 4). Each bin width: 20 ms. Error bars indicate the standard error of the mean. **(D)** The mean z-score of the number of successful attempts per lag interval in mice that experienced sessions with the link plate. A significant difference was noted between the lag interval of <20 ms and other lag intervals (Factor: Lag, Levels: 50, *F*-value: 16.6, Degrees of freedom: between = 49 within = 250, Adjusted *p* = $1.07\times 10^{-7}$ ,one-way analysis of variance [ANOVA], Tukey–Kramer test, *p < 0.05; *n* = 6). The adjusted *p*-value corresponds to the comparison with the lag interval of 20–40 ms. Each bin width is20 ms. The error bars indicate the standard error of the mean. **(E)** The mean z-score of the number of successful attempts per lag interval in mice that did not experience sessions with the link plate. No significant difference was found between the <20 ms and 20–160 ms lag intervals (Factor: Lag, Levels: 50, F-value: 10.5, Degrees of freedom: between = 49 within = 150, Adjusted *p* = 1, one-way ANOVA, Tukey–Kramer test; *n* = 4). The adjusted *p*-value corresponds to the comparison with the lag interval of 20–40 ms. Each bin width is 20 ms. The error bars indicate the standard error of the mean.

To evaluate the effectiveness of experiencing the sessions with the link plate, we compared data on mice that experienced these sessions (*n* = 7) and on those that did not experience them (*n* = 4). First, we compared the success rate of the first independent session. The success rate of mice that experienced the sessions with the link plate tended to be higher than that of mice that did not experience them, however the difference was not statistically significant (*p* = 0.31, unpaired t-test, Supplementary Figure 4). Next, we compared the lag in the independent sessions. We defined the lag as the time difference between the onset of the right and left lever pulls (Figure 4A). We calculated the mean number of successful attempts within each lag interval (each bin width: 20 ms) across the independent sessions produced a histogram with the values at sessions 13–15 (Figures 4B,C). The mean number of successful attempts with a lag interval of ≥220 ms was smaller than the mean number of successful attempts with a lag interval of 200–220 ms (data not shown in Figures 4B,C). The mean number of successful attempts with a lag interval of <20 ms was 50.4 for the mice that had experienced the sessions with the link plate (Figure 4B), compared to 13.6 for those that did not experience these sessions with the link plate (Figure 4C). To standardize the assessment of the relative frequency of the number of successful attempts across lag intervals, irrespective of the absolute number of successful attempts, the number of successful attempts included in each lag interval was converted into z-scores within each session. The mean z-score was calculated across the independent lever sessions (Figures 4D,E). Most of the mean z-scores of the number of successful attempts with a lag interval of ≥220 ms were less than zero; thus, these data are not shown in Figures 4D,E. A significant difference was noted between the lag interval of <20 ms and those of ≥20 ms in the mice that had experienced sessions with the link plate (Figure 4D). In contrast, in the mice that did not experience sessions with the link plate, a similar lag interval comparison was performed, but no significant difference was observed between the lag intervals of <20 ms and 20–160 ms intervals (Figure 4E). Furthermore, in the population analysis of mice that did not experience sessions with the link plate, no significant differences were observed in success rate, response latency, or number of successful attempts as the number of sessions increased (Supplementary Figures 2B–E). These results indicate that it is harder for mice to learn the simultaneous lever pulls without using the link plate. These findings signify that mice that underwent sessions with the link plate exhibited a higher proportion of simultaneous lever pulls with lag intervals of <20 ms than the mice that did not undergo these sessions, indicating that training in the linked state facilitated simultaneous bimanual lever-pull movements.

## 4 Discussion

In this study, we successfully developed a novel experimental system for assessing simultaneous bimanual movements in head-fixed mice. In this task, mice were trained to pull the right and left levers simultaneously with bilateral forelimbs in response to a sound cue. This task was achieved by introducing sessions with the link plate where both levers were mechanically linked and could be moved simultaneously, helping the mice grasp the importance of simultaneous bimanual movements for obtaining rewards. These sessions with the link plate enabled the mice to maintain a high success rate even during the independent state of the levers. Consequently, the mice that experienced sessions with the link plate showed a significantly higher ratio of simultaneous lever pulls with a lag within 20 ms than nonsimultaneous lever pulls (Figure 4D). In contrast, mice without session with the link plate experience exhibited no significant increase in simultaneous pulls over nonsimultaneous ones (Figure 4E). These results demonstrate that experience with the sessions with the link plate provided effective training for performing simultaneous bimanual movements. To our knowledge, this is the first task developed for repetitive simultaneous movements of bilateral forelimbs in head-fixed rodents. Because M1 exhibits a contralateral preference for body parts, bimanual movements require coordinated information processing between both cortical hemispheres (Donchin et al., 1998; Kazennikov et al., 1999; Cross et al., 2020) via the corpus callosum (Diedrichsen et al., 2003; Tazoe et al., 2013; Gooijers and Swinnen, 2014; Jeong et al., 2021). Furthermore, bimanual coordinated movements require the involvement of multiple cortical areas, including the supplementary motor area (Tanji et al., 1987, 1988; Sadato et al., 1997; Kazennikov et al., 1999; Aramaki et al., 2010) and dorsal premotor area (Sadato et al., 1997; Kermadi et al., 2000; Aramaki et al., 2010). Analysis of the simultaneous bimanual movement task for head-fixed rodents by calcium imaging and electrophysiological techniques facilitates the investigation of the dynamics of neural activity, particularly that in the callosal axons and motor cortex areas. Therefore, the task we developed helps elucidate the pathophysiology of impaired bimanual movements and facilitates the development of novel therapeutic and rehabilitation strategies (Kantak et al., 2017).

Complex movements consist of various motor elements, including unimanual, simultaneous, and nonsimultaneous bimanual movements. Revealing and comparing neural activities associated with each movement element would elucidate the information processing mechanisms of complex movements (Flash and Hochner, 2005; Tresch and Jarc, 2009; Bizzi and Cheung, 2013; Giszter, 2015; Miranda et al., 2018). While previous studies have investigated these motor elements separately (Isomura et al., 2009; Hira et al., 2013; Masamizu et al., 2014; Soma et al., 2017; Jeong et al., 2021; Terada et al., 2022), direct comparisons of neural activities during transitions between these elements within the same animal remain unexplored. Unlike sequential bimanual movement, simultaneous movement may require more coordinated neural processing between both hemispheres to strictly synchronize both limbs strictly. The task developed in this study may help determine the neural mechanisms underlying simultaneous bimanual movements by enabling comparisons of the neural circuits of simultaneous and nonsimultaneous lever pulls. The use of a device that controls the type of lever movements is instrumental for ensuring a seamless transition between different movements, such as simultaneous, unimanual, and sequential movements. Combining our method with different cues indicating different task types, such as unimanual and bimanual movements, would enable studies of different types of tasks performed by the same animal. For example, it is already known that mice can discriminate between different tone cues (Kondo and Matsuzaki, 2021). In future experiments, the same mice will be trained to perform bimanual lever pulls in response to one tone and unimanual lever pulls in response to a different tone. Furthermore, by comparing brain activities during these tasks, we could elucidate the significance of bimanual movements.

The potential of several parameters used in this study to facilitate task learning needs to be discussed. For example, the lever displacement threshold was fixed at 40% of the maximum in this study. This value was selected based on previous studies performed using similar lever-pull tasks in head-fixed mice. For example, Terada et al. (2022) employed a displacement threshold of 20% in a sound-cued lever-pull task, while Shinotsuka et al. (2023) used a displacement threshold of 50% for a spontaneous lever-pull task. After looking at these two studies, we opted for a displacement threshold of 40%. However, selecting a lower displacement threshold during the initial phase of training might facilitate task learning of the task. Another parameter is the allowable lag between the right and left lever pulls. In this study, we limited the latency of both lever pulls to 1 s but did not limit the duration of the lag to prevent discouraging the animals’ task learning. This method combined with the experience of sessions with the link plate helps the animals learn the task successfully (Figure 4). Although these results indicate the effectiveness of experiencing sessions with the link plate, it is not ruled out whether limiting the allowable lag enhances the task learning. For example, if rewards were given only when mice pulled the right and left levers within a 20-ms lag, mice that had not undergone the session with the link plate also could have shown a higher success rate. We argued about the increase of divergence between the levers in the independent session, especially after the reward (Figures 3A,B). In this method, we did not control the timing of lever recovery; however, it might be possible to control this timing as well as lever-pull if it is triggered by another sound cue. Therefore, these parameters need to be carefully selected so that animals can learn effectively in future studies.

In conclusion, our method enables direct comparisons of brain activity dynamics, such as those in the callosal axons and motor cortex regions and across various motor elements via calcium imaging and electrophysiology. Overall, our developed task significantly facilitates understanding of complex movement processing by providing a robust framework for studying the neural mechanisms underlying simultaneous bimanual movements.

## Data Availability

The raw data supporting the conclusions of this article will be made available by the authors, without undue reservation.
