# Supplementary material for: Effective training procedure for a simultaneous bimanual movement task in head-fixed mice
Source: Front Neural Circuits. 2025 Aug 8;19:1633843. doi: 10.3389/fncir.2025.1633843 (PMC12370711; doi:10.3389/fncir.2025.1633843)
Supplement: Supplementary file 1 [file Data_Sheet_1.PDF]

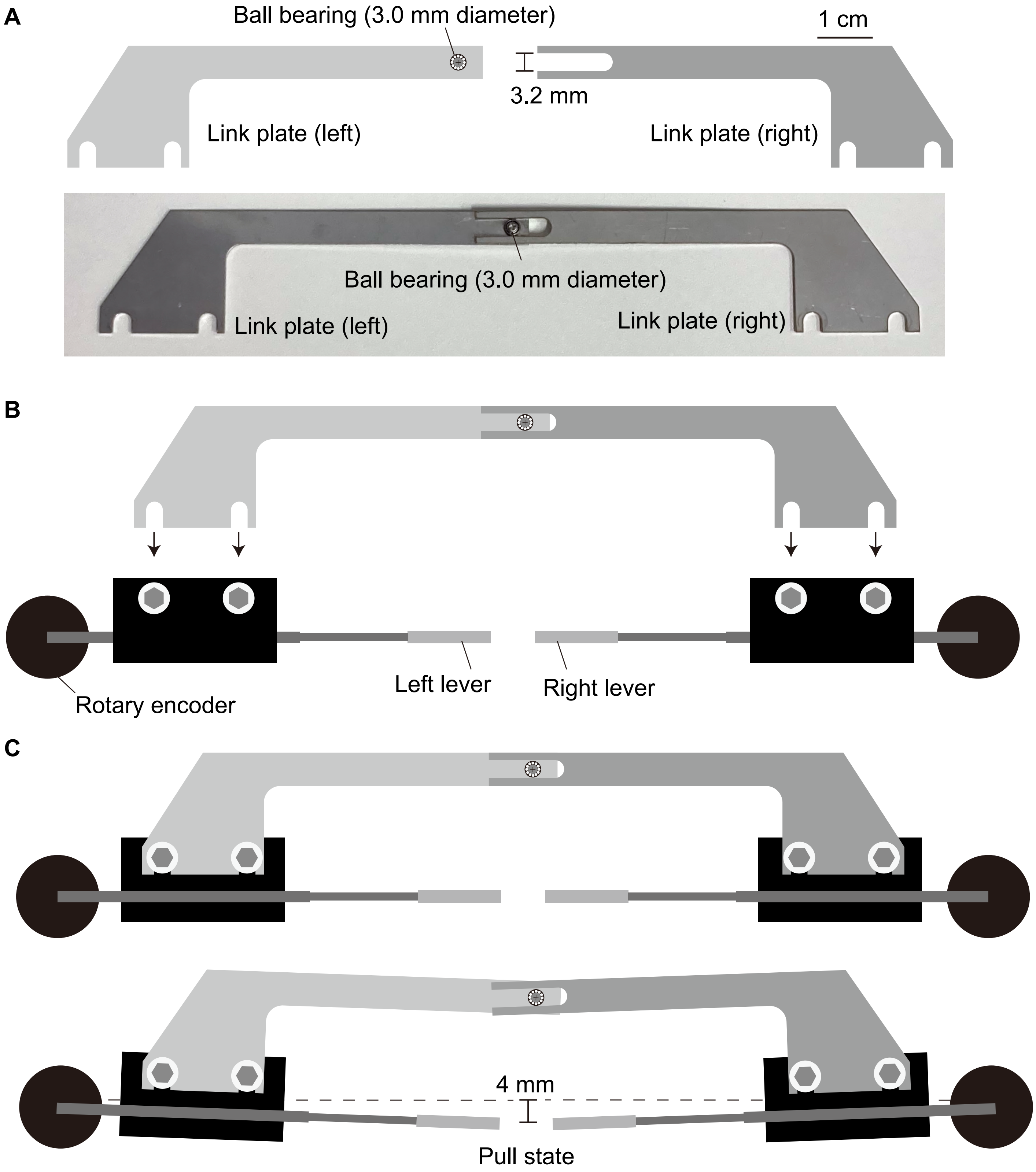

Supplementary Figure 1. The link plate apparatus.

(A) Top, illustration of the link plate. The link plate can be separated into the right and left plate. There is 0.2 mm of mechanical lash between the ball bearing on the left plate and the hole on the right plate. Bottom, a photograph of the link plate.

(B) The link plate can be attached to the lever shaft by the cap screws.

(C) When the link plate is attached to the levers, both levers move synchronously.

A

Session	Licking sessions		Preparatory sessions	Independent sessions
	1	2	3 sessions	1–10
**Task type**	Licking		Lever-pull	
**Lever pull time**			1–400 ms	400 ms
**Lever state**			Independent	

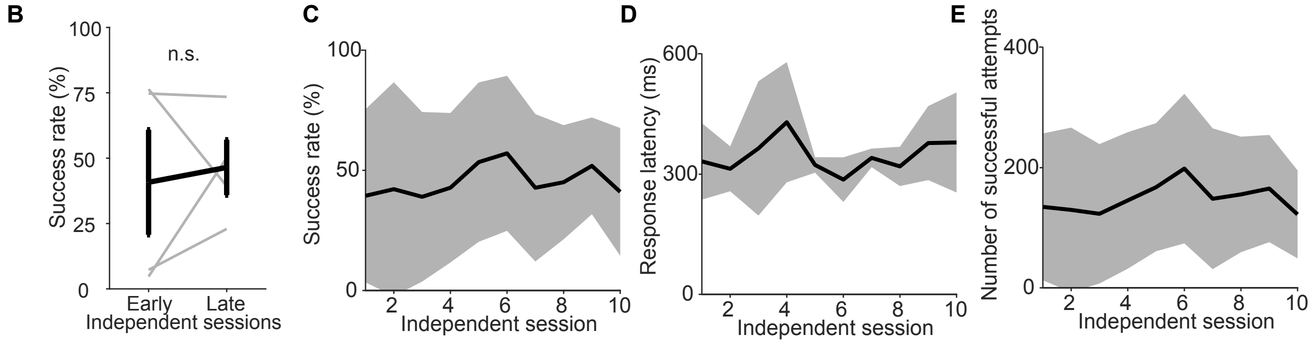

Supplementary Figure 2. Mice that did not experience sessions with link plate.
(A) Training schedule of the sound cue-triggered simultaneous bimanual lever-pull task of mice that did not experience sessions with link plate.
(B) Changes in the success rate during independent sessions 1–2 (early) and 9–10 (late). Gray lines indicate individual mice. The bold black line indicates the mean success rate among the mice. No significant difference in success rate was observed between the early and late periods ($p = 0.88$, Wilcoxon signed-rank test; $n = 4$).
(C) Changes in the success rate of the population means. The black line and gray area indicate the mean and standard deviation, respectively. The bars indicate the number of trials. There was no significant change in success rate across sessions (GLMM, $p = 0.36$; β_1 ± standard error was 0.71 ± 0.062; exp(β_1) was 1.07 (95% CI, 0.95–1.22); β_0 was 3.19; $n = 4$).
(D) Changes in the response latency of the population means. The black line and gray area indicate the mean and standard deviation, respectively. The response latency significantly decreased across sessions (GLMM, $p = 0.078$; β_1 ± standard error was 0.0065 ± 0.024; exp(β_1) was 1.01 (95% CI, 0.96–1.06; β_0 was 5.80; $n = 4$).
(E) Changes in the number of successes of the population means. The black line and gray area indicate the mean and standard deviation. There was no significant difference in the number of successes (GLMM, $p = 0.19$; β_1 ± standard error was 0.072 ± 0.055; exp(β_1) was 1.08 (95% CI, 0.96–1.20); β_0 was 4.31; $n = 4$).

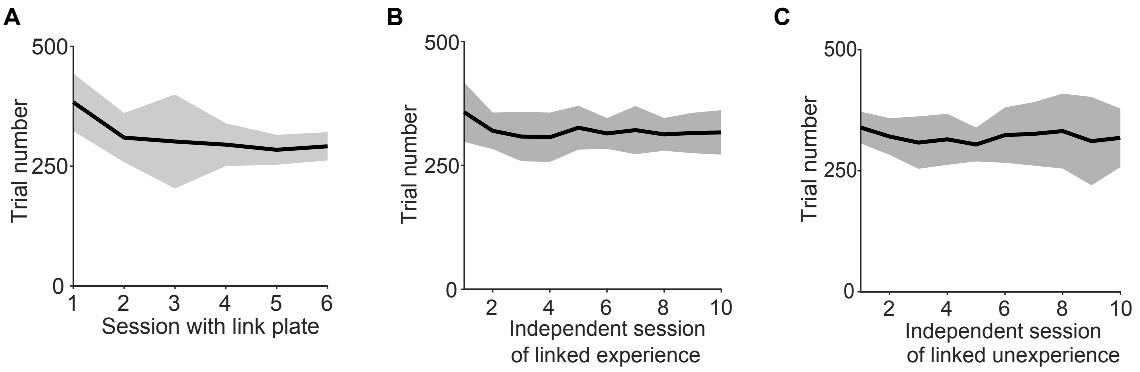

Supplementary Figure 3. The trial number of mice that experienced sessions with link plate and those that did not experience them.

(A) Changes in the trial number of the population means in sessions with link plate of mice that experienced sessions with the link plate. The black line and gray area indicate the mean value and standard deviation, respectively (n = 7).

(B) Changes in the trial number of the population means in independent session of mice that experienced sessions with the link plate. The black line and gray area indicate the mean value and standard deviation, respectively (n = 7).

(C) Changes in the trial number of the population means in independent session of mice that did not experience sessions with the link plate. The black line and gray area indicate the mean value and standard deviation, respectively (n = 4).

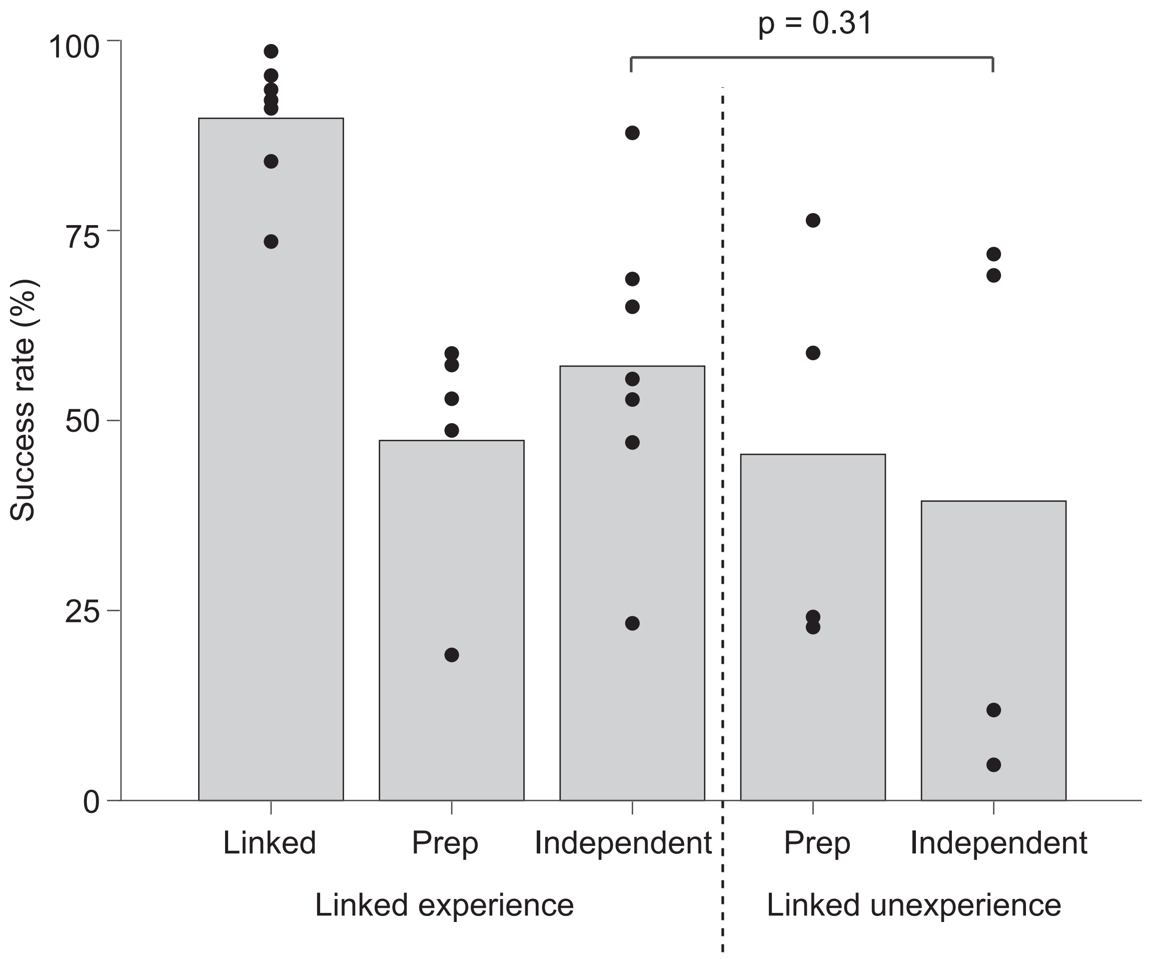

Supplementary Figure 4. Comparison between linked-experienced and linked-unexperienced mice. The success rates for the following sessions: the last linked session, the last preparatory session, and the first independent session in mice that experienced the sessions with the link plate (n = 7); and the last preparatory session and the first independent session in mice that did not experience the sessions with the link plate (n = 4). The success rate in the first independent session was lower in mice that did not experience sessions with the link plate; however, the difference was not statistically significant (p = 0.31, unpaired t-test). As two mice that experienced sessions with link plate did not undergo preparatory sessions, their data were excluded from analysis of preparatory sessions.
