# Supplementary material for: Effective training procedure for a simultaneous bimanual movement task in head-fixed mice
Source: Front Neural Circuits. 2025 Aug 8;19:1633843. doi: 10.3389/fncir.2025.1633843 (PMC12370711; doi:10.3389/fncir.2025.1633843)
Supplement: Supplementary file 2 [file Data_Sheet_2.PDF]

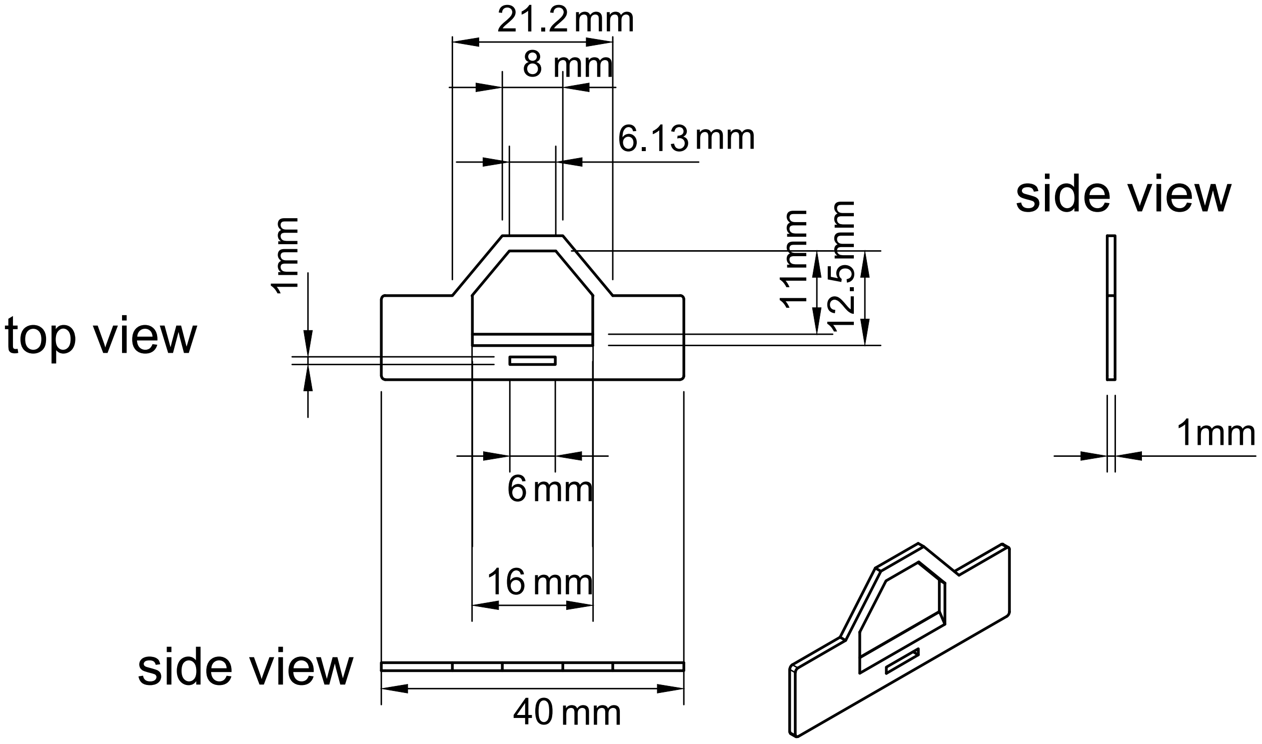


| Dept. | Technical reference | Material Stainless Steel | 2.9 g |
|---|---|---|---|
| | | Document type | Document status |
| | | Title Headplate Tezuka et al. | DWG No. |
| | | | Rev. / Date of issue / Sheet 1/1 |
